# Growth Inhibitory Effect of Garlic Powder and Cinnamon Extract on White Colony-Forming Yeast in Kimchi

**DOI:** 10.3390/foods10030645

**Published:** 2021-03-18

**Authors:** Mi-Ju Kim, Seong-Eun Kang, Chang Hee Jeong, Sung-Gi Min, Sung Wook Hong, Seong Woon Roh, Deok-Young Jhon, Tae-Woon Kim

**Affiliations:** 1 Research and Development of Division, World Institute of Kimchi, Gwangju 61755, Korea; mijukim79@gmail.com (M.-J.K.); seongeun328@gmail.com (S.-E.K.); jeongch@wikim.re.kr (C.H.J.); skmin@wikim.re.kr (S.-G.M.); swhong@wikim.re.kr (S.W.H.); swroh@wikim.re.kr (S.W.R.); 2 Department of Food and Nutrition, Chonnam National University, 77, Yongbong-ro, Buk-gu, Gwangju 61186, Korea; dyjhon@jnu.ac.kr

**Keywords:** kimchi, white colony-forming yeast, anti yeast activity, garlic, cinnamon

## Abstract

White colony-forming yeast (WCFY), also referred to as film forming yeast or spoilage yeast, that appear on the surface of kimchi can deteriorate the sensory properties of kimchi, such as odor and texture. Thus, the aim of this study was to develop a method to inhibit the formation of the white colony in kimchi. First, alterations in kimchi manufacturing and storage conditions, including temperatures, pH, salinity, and anaerobic condition, were investigated to determine if they could inhibit the growth of WCFY (i.e., *Kazachstania servazzii*, *Candida sake*, *Debaryomyces hansenii*, *Pichia kudriavzevii*, and *Hanseniaspora uvarum*). Thereafter, the anti yeast activity of freeze-dried garlic powder (FGP) and cinnamon ethanol extract (CEE) was evaluated against WCFY using the agar-well diffusion assay. Following the direct application of FGP and CEE to the surface of the kimchi, the inhibitory effects on white colony were determined. The results showed that WCFY can grow under various manufacturing and storage conditions of kimchi. Regarding the growth inhibitory effect on WCFY, FGP exhibited anti yeast activity against four WCFYs. It did not show anti yeast activity against *K. servazzii*. However, CEE showed anti yeast activity against *K. servazzii*. In particular, the mixture of 10% FGP and 1.75% CEE, which was manufactured considering the influence of sensory properties in kimchi, exhibited anti yeast activity against all WCFY. Furthermore, the application of the FGP and CEE mixture supplemented with 0.02% xanthan gum to kimchi to enhance adhesion to the kimchi surface, led to a delay in the formation of a white colony on the surface of the kimchi by an average of 17 d at 10 °C compared to the control group. Collectively, the use of a FGP, CEE, and xanthan gum mixture could be an effective method for the inhibition of white colony formation on the surface of kimchi, extending its shelf life.

## 1. Introduction

Kimchi, a traditional Korean fermented vegetable product, is produced via the fermentation of salted vegetables with various spices and other ingredients including garlic, red pepper powder, and ginger. The fermentation of kimchi is initiated by various microorganisms originally present in the raw materials [1]. It is well established that lactic acid bacteria including *Leuconostoc* spp., *Weissella* spp., and *Lactobacillus* spp. play an important role during kimchi fermentation [2]. However, during kimchi fermentation and storage, the growth of white colony-forming yeast (WCFY), also called spoilage yeast or film forming yeast, can occur on the surface of kimchi and can lead to the deterioration of the quality of the kimchi, such as an off-odor production and texture-softening [3,4,5,6]. Thus, the formation of an undesirable white colony on kimchi can lead to enormous economic loss in the commercial kimchi market, especially the export market, which requires long transportation. However, studies on the inhibition of white colony on the surface of kimchi have rarely been reported and only a few technologies have been practically applied to the kimchi.

The inhibition of spoilage yeasts is one of the most important aspects of food preservation. To control spoilage yeasts, several methods ranging from chemical to physical preservation techniques have been adopted by the food industry [7,8,9]. Chemical preservatives such as potassium sorbate, sodium metabisulfite, and sodium benzoate have been widely used in the preservation of acidified vegetables [10]. However, numerous consumers consider the use of these chemical preservatives in foods to be undesirable and their use in kimchi is constrained by legal limitations. In the Korean Food Code, the application of chemical preservatives to kimchi is prohibited, and they is also not permitted as food additives in the CODEX (Codex Alimentarius Commission) standard for kimchi. Moreover, thermal treatments lead to the loss of sensory properties and the nutritional attributes of kimchi. Therefore, it is necessary to develop a method for inhibiting the growth of WCFY in kimchi using natural substances while considering cost and sensory properties. Various natural preservatives, such as green tea seed extract [11], grape seed extract [12], horse-radish powder [13], and bran of oat extract [14], have been applied to different food products to elicit an antimicrobial effect. Among them, garlic and cinnamon have been extensively studied for their antimicrobial activities [15,16,17,18]. According to a previous study, allicin, a main compound in crushed garlic, exerted broad antimicrobial activities against different bacteria and fungi [19]. Additionally, cinnamaldehyde, a major component in cinnamon extract, demonstrated an antifungal effect in both bread and cheese during storage [20].

Therefore, in this study, we investigated the growth characteristics of five yeast strains to determine whether the commercial conditions of kimchi fermentation and storage inhibit white colony formation. Moreover, the anti yeast activity of freeze-dried garlic powder (FGP) and cinnamon ethanol extract (CEE) were evaluated against WCFY to develop a new preservation method for kimchi.

## 2. Materials and Methods

### 2.1. Yeast Strains

A total of five yeast strains reported as major yeasts associated with undesirable white colony formation on the surface of kimchi were used in this study [3,5,6]. *Kazachstania servazzii* MGB0660, *Candida sake* MGB0659, and *Debaryomyces hansenii* MGB0661 were directly isolated from white colonies that appeared on the surface of kimchi, and *Pichia kudriavzevii* MGB1001 and *Hanseniaspora uvarum* MGB1002 were isolated from red pepper powder, which is an ingredient of kimchi. Briefly, white colonies from kimchi surface were picked and plated on yeast extract-peptone-dextrose (YPD) agar (Difco, Sparks, MD, USA). Red pepper powder was 10-fold diluted with sterile water, and 0.1 mL of the dilution was plated on YPD agar. Then, all agar plates were incubated at 24 °C for 48 h. To investigate yeasts associated with white colony formation, isolates were separately inoculated into kimchi and were selected, causing white colony formation [3]. The isolates were identified by amplifying the internal transcribed spacer (ITS) region of yeast using the universal primer pair ITS1F (5′-CTTGGTCATTTAGAGGAAGTAA-3′) and ITS4 (5′-TCCTCCGCTTATTGATATGC-3′) as previously reported [21]. Amplicons were sequenced with the ABI PRISM 3700 DNA Analyzer (Life Technologies, Carlsbad, CA, USA), and sequences were identified using a BLAST search against the UNITE database (https://unite.ut.ee/, accessed on 10 March 2021).

### 2.2. Growth of WCFY under Different Conditions

The growth rate of WCFY was measured under different conditions of temperature, pH, salinity, and anaerobic culture while considering the manufacturing and storage conditions of kimchi. First, each yeast strain was inoculated into YPD broth and incubated at 0, 4, 10, and 20 °C for 19, 15, 5, and 4 days, respectively. In addition, WCFY was inoculated into YPD broth adjusted to pH 3, 4, and 5 with hydrochloric acid and cultured at 24 °C for 120 h. The growth ability of WCFY was measured at an optical density (OD) of 600 nm using an Infinite M200 PRO microplate reader (Tecan, Mannedorf, Switzerland) and performed in triplicate [4]. Thereafter, the growth of WCFY was also examined under various salinities (10, 15, and 20% (*w/v*) NaCl-containing media) using YPD agar media. For yeast counts, each WCFY dilutions was inoculated onto YPD agar media and incubated at 24 °C for 48 h. For the anaerobic growth test, each yeast strain was cultured anaerobically on YPD agar media at 4, 10, and 20 °C using the GasPak EZ Anaerobe Gas Generating Pouch System (BD Biosciences, Sparks, MD, USA).

### 2.3. Preparation of FGP and CEE 

FGP was obtained from Namhae Garlic Research Institute in South Korea. FGP was dissolved in sterilized water at concentrations of 10, 20, and 30% (*w/v*). Cinnamon powder was purchased from TRI Co., Ltd. (Eumseong, Korea). To manufacture cinnamon water extract, 10 g of cinnamon powder was soaked in 40 mL of water at 4 or 60 °C for 24 h. To manufacture CEE, 75 g of cinnamon powder was soaked in 30, 50, 80, or a 100% concentration of 300 mL ethanol at room temperature (23–26 °C) for 24 h. The extract was centrifuged at the speed of 3900 rpm for 10 min, and the supernatant was collected and stored at 4 °C until used.

### 2.4. Analysis of Alliin and Cinnamaldehyde Content 

Each sample was prepared as follows: 5 mL of sample was centrifuged at 4 °C (13,000 rpm for 5 min), and the supernatant was filtrated using a 0.2 μm syringe filter (Sartorius Stedim Biotech GmbH, Göttingen, Germany). The alliin level in FGP and raw garlic filtrate was measured using a Shimadzu liquid chromatography-mass spectrometry (LC-MS) 8050 triple-quadrupole mass spectrometer (Shimadzu, Kyoto, Japan) equipped with an electrospray ion source as described previously [22]. Briefly, the chromatographic separation was performed using a Restek Ultra PFPP column (3 μm, 150 × 2.1 mm; Restek, Bellefonte, PA, USA) with an Ultraguard (5 μm, 10 × 2.1 mm; Restek, Bellefonte, PA, USA). The binary gradient system was comprised of (A) water supplemented with 0.1% formic acid and (B) 0.1% acetonitrile. The mobile phase was programmed as follows: 0–15 min, (B) 0–100%; 15–25 min, (B) 100%. The injection volume was 1 μL and the flow rate was set at 0.25 mL/min. The temperature of the column oven was maintained at 30 °C. 

The cinnamaldehyde level in CE was measured using the Ultimate 3000 high performance liquid chromatography (HPLC) system with ultraviolet–visible (UV/Vis) spectrophotometric detector (Thermo Fisher Scientific, Waltham, MA, USA) as described previously with some modifications [23]. Briefly, the chromatographic separation was conducted using an Inno C-18 column (5 μm, 4.6 × 250 mm; Youngjin Biochrome, Seongnam-si, Korea). The mobile phase was a mixture of water:methanol (40:60; *v/v*). The injection volume was 10 μL and the flow rate was set at 1.0 mL/min. The temperature of the column oven was maintained at 30 °C. The UV detector was set at 290 nm.

### 2.5. Anti Yeast Activity of FGP, CEE, and the FGP and CEE Mixture

The anti yeast activity of FGP, CE, and their mixture were evaluated using an agar-well diffusion assay with some modifications [24]. Briefly, WCFY were cultured in YPD broth at 24 °C for 24–48 h (up to 7 log CFU/mL). A total of 70 µL of each yeast strain was inoculated in 7 mL sterilized YPD soft agar and poured onto a YPD agar plate. Thereafter, FGP, CE, and their mixture were added to each well (8 mm in diameter) on the agar plate and incubated at 24 °C for 24–48 h. The antagonistic effect of FGP, CE, and their mixture on WCFY was determined by each clear zone.

### 2.6. Sensory Analysis

A sensory test was conducted by 10 panelists (5 male and 5 female) to determine the difference between kimchi samples treated with the mixture of FGP and CEE using a 9-point hedonic scale [25,26]. Appearance, smell, and taste were included as indicators. Samples were served in a paper cup identified by a three-digit code. Panelists were asked to select a score from 1 (extremely bad) to 9 (extremely excellent) for each kimchi sample. They also used water to cleanse their palates between each sample evaluations. 

### 2.7. Application of the FGP and CEE Mixture in Kimchi

To evaluate the growth inhibitory effect of the FGP and CEE mixture against WCFY, each WCFY strain was inoculated into kimchi, which was subsequently treated with the mixture. The experiment was conducted as previously described with some modifications [10]. Kimchi was ground using a hand blender (Philips, Amsterdam, The Netherlands) and autoclaved at 121 °C for 15 min to remove yeasts that were originally present in the kimchi. Thereafter, 200 μL of WCFY culture broth (5 log CFU/mL) was inoculated into 20 g of kimchi sample, and 3 g of sample including the control group (only culture broth without WCFY) were placed into each well in 12-well plate. Thereafter, the FGP and CEE mixture was applied to the kimchi inoculated with WCFY and stored at 24 °C for 10 day. 

Moreover, to determine the applicability of the mixture of FGP, CEE, and xanthan gum (ES Food, Gunpo, Korea) in commercial kimchi, it was sprayed onto the surface of kimchi in a plastic container and stored at 10 °C. The mixture was sprayed onto kimchi samples (0.03 g/cm^2^) and observed in storage until a white colony was formed. 

### 2.8. Statistical Analysis

Data are represented as the mean ± standard error (SE). Statistical significance was determined using the Predictive Analytics Software (PASW) statistics for Windows, version 19.0 (SPSS, Chicago, IL, USA). A *p* value < 0.05 was considered statistically significant difference. 

## 3. Results

### 3.1. Growth Ability of WCFY under Different Cultivation Conditions

In this study, five genera of yeasts, such as *Kazachstania*, *Candida*, *Debaryomyces*, *Pichia*, and *Hanseniaspora*, were selected, because they were reported as major yeasts associated with undesirable white colony formation on the surface of kimchi [3,4,5]. We isolated five yeast strains of *K. servazzii*, *C. sake*, *D. hansenii*, *P. kudriavzevii*, and *H. uvarum* from kimchi and a red pepper powder used as a kimchi ingredient. We evaluated the growth characteristics of these yeasts under various manufacturing and storage conditions of kimchi.

#### 3.1.1. Temperature

**Figure 1:**
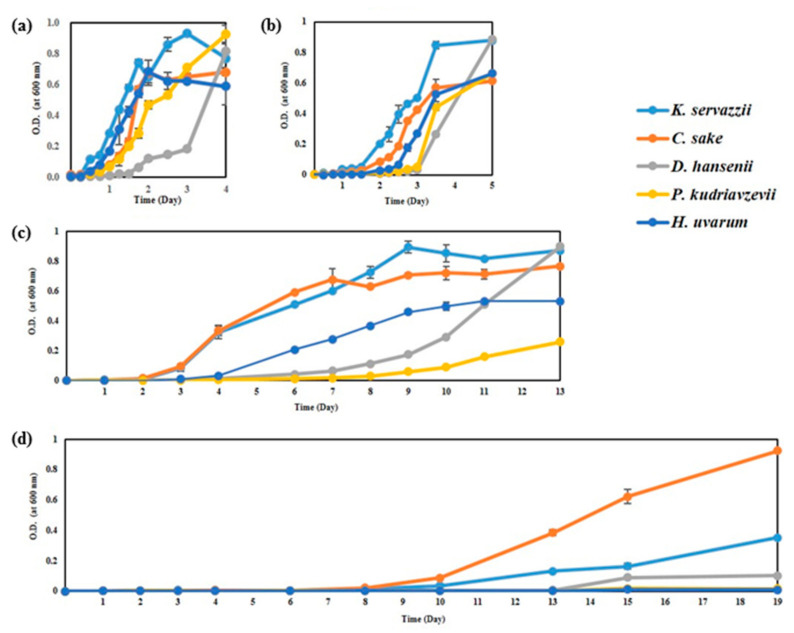
Growth curve of five white colony-forming yeast (WCFY) at different temperatures of (**a**) 20, (**b**) 10, (**c**) 4, and (**d**) 0 °C. O.D. means optical density.

The growth ability of WCFY was evaluated while considering kimchi fermentation and storage temperatures (0, 4, 10, and 20 °C). Although there were differences in growth rates between yeast strains, all WCFY grew at 4, 10, and 20 °C (Figure 1). However, only *K. servazzii*, *C. sake*, and *D. hansenii* grew at 0 °C (Figure 1d). According to our results, WCFYs are able to grow at commercial storage temperature (0–10 °C), causing undesirable white colony formation in kimchi during storage.

#### 3.1.2. pH

**Figure 2:**
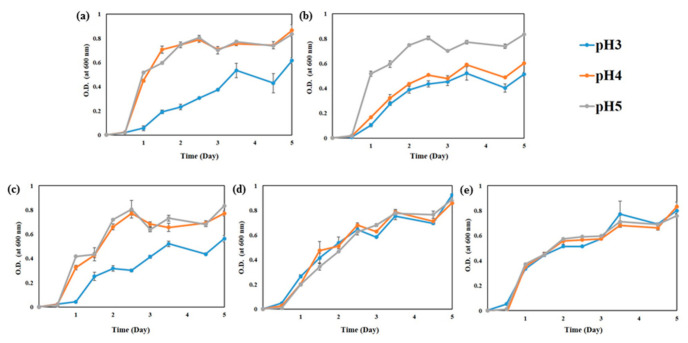
Growth curve of five white colony-forming yeast (WCFY) at pH 3, 4, and 5. (**a**) *Kazachstania servazzii*; (**b**) *Candida sake*; (**c**) *Debaryomyces hansenii*; (**d**) *Pichia kudriavzevii*; (**e**) *Hanseniaspora uvarum*.

The pH of kimchi at the initial fermentation stage is approximately 5.0 and gradually decreases to below pH 4.0 during fermentation. Thus, the growth ability of WCFY was estimated at pH 3, 4, and 5. Although the growth of *K. servazzii*, *C. sake*, and *D. hansenii* was slightly late compared to *P. kudriavzevii* and *H. uvarum*, all WCFY grew well at pH 3, 4, and 5 (Figure 2). These results explain why white colony was well formed in over-ripened kimchi despite low pH.

#### 3.1.3. Salinity

**Figure 3:**
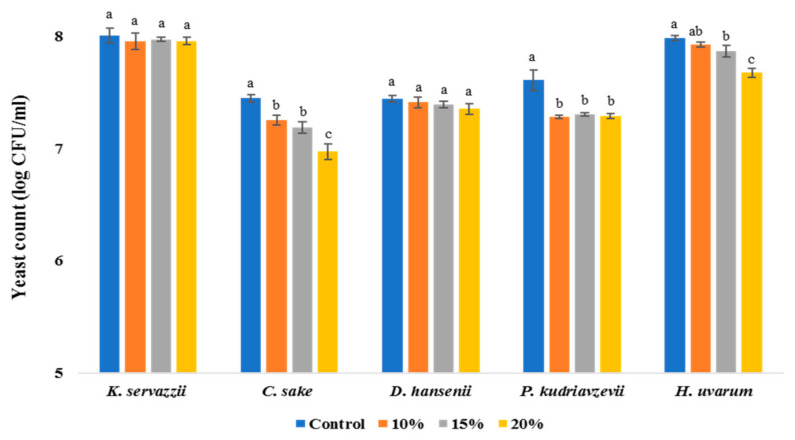
Growth of five white colony-forming yeast (WCFY) under different salinity of 10, 15, and 20%. Values are mean ± standard error (*n* = 3). Different letters mean significant differences (*p* < 0.05).

Given that kimchi cabbages were commonly salted in water containing approximately 10–15% (*w/v*) salt prior to kimchi manufacture, the viability of WCFY was evaluated at salinities of 10, 15, and 20%. In Figure 3, as salinity increased, the viable cell counts of *C. sake*, *P. kudriavzevii*, and *H. uvarum* significantly decreased. However, even at the high salinity of 20%, all WCFY survived (Figure 3). Therefore, the high-salt water used to produce kimchi did not have a significant effect on inhibiting white colony formation on kimchi. Similarly, a previous study also showed that salt did not have a significant effect on the growth inhibition of *Pichia membranifaciens*, spoilage yeast during kimchi fermentation [27].

#### 3.1.4. Anaerobic Conditions

To determine the effect of anaerobic conditions on the appearance of WCFY in kimchi, five yeast strains were cultured under anaerobic conditions at 4, 10, and 20 °C. These colonies were observed at all incubation temperatures (data not shown). At 4 °C, the *P. kudriavzevii* colony was formed late in a small size compared with different temperature conditions. It is thought that this is the reason as to why white colony formed on the surface of fermented kimchi whose packaging has not been opened despite the relative anaerobic environment due to the carbon dioxide produced by lactic acid bacteria during fermentation [28,29]. Although oxygen exposure has been known as one of the important factors that promote white colony formation on the surface of kimchi [6], the limitation of oxygen exposure was not enough to inhibit the appearance of WCFY on kimchi.

### 3.2. Anti Yeast Activity of Garlic and Cinnamon against WCFY

**Figure 4:**
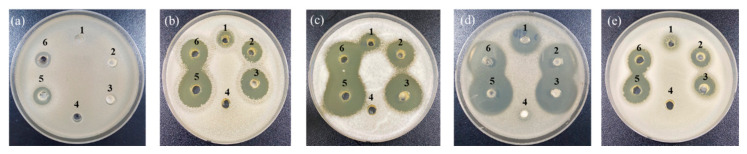
Anti yeast activity of various garlic extracts against (**a**) *Kazachstania servazzii*, (**b**) *Candida sake*, (**c**) *Debaryomyces hansenii*, (**d**) *Pichia kudriavzevii*, and (**e**) *Hanseniaspora uvarum* by agar-well diffusion assay: 1–3, 10, 20, and 30% (*w/v*) of freeze-dried garlic powder; 4, garlic concentrate; 5, raw garlic filtrate; 6, raw garlic filtrate after storage of 2 days.

In a previous study, garlic demonstrated antimicrobial activity against *Salmonella enteritidis*, *Staphylococcus aureus*, *Escherichia coli*, *Bacillus cereus*, and *Listeria monocytogenes* [30]. Particularly, among the compounds derived from garlic, alliin is a representative substance that contributes most to the flavor of garlic and is the precursor of thiosulfinates [31]. Moreover, when garlic is cut or crushed, allicin is formed from alliin by alliinase [32]. Allicin has been known to exert high antifungal and antibacterial activity [33]. Thus, the growth inhibitory effect of garlic against WCFY was evaluated using an agar-well diffusion assay. Raw garlic filtrate and FGP showed strong anti yeast activity against four WCFYs except *K. servazzii* (Figure 4). *K. servazzii* was marginally inhibited only by raw garlic filtrate, and not by FGP (Figure 4a). In contrast, garlic concentrate did not demonstrate any anti yeast activity against the WCFYs tested. The inhibition zone of garlic concentrate was not observed in the agar-well diffusion assay (Figure 4). These results are presumed to be caused by the high temperature inactivating alliinase in garlic concentrate during processing and alliin could not be converted into allicin. According to a previous study, the antimicrobial activity of garlic decreased as the heat treatment temperature increased [34]. However, in order to accurately determine the anti yeast activity of garlic concentrate, further studies on the measurement of alliin content and alliinase activity are required. Thereafter, the alliin content in FGP and raw garlic filtrate was measured using LC-MS analysis (Table 1). As a result, the alliin content of FGP increased in a concentration-dependent manner, and the size of the inhibition zones also increased as the powder concentration increased. In general, after white-colony formation, the yeast colonies spread gradually on the surface of the kimchi (white-film formation) and eventually led to an off-odor and texture softening. Natural preservatives in the kimchi industry should be sprayed on the surface of the kimchi because WCFY is formed there rather than on the part with kimchi soup. Furthermore, spraying the preservatives on the surface is more appropriate than mixing them with seasoning in terms of the cost and sensory properties. A previous study reported that spraying cinnamon and clove essential oils could reduce the development of mold spoilage in a wheat bread sample during the storage period [15]. Our results showed that the inhibitory effect of the raw garlic filtrate was higher than that of FGP on all WCFY strains even though 30% FGP had more alliin than raw garlic filtrate (Figure 4, Table 1). It was assumed that the activity of the alliinase was reduced during the drying process of the garlic powder [35]. However, the raw garlic filtrate was difficult to apply to kimchi industrially because it was sticky and perishable, and had a strong odor. Thus, commercially processed FGP might be used in owing to its ease of use as an alternative to raw garlic filtrate.

**Figure 5:**
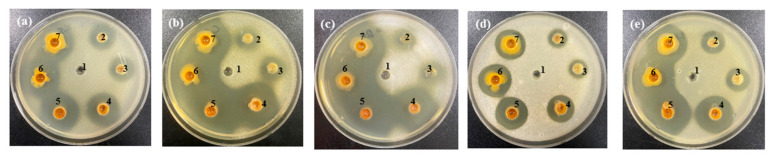
Anti yeast activity of cinnamon extract (CE) against (**a**) *Kazachstania servazzii*, (**b**) *Candida sake*, (**c**) *Debaryomyces hansenii*, (**d**) *Pichia kudriavzevii*, and (**e**) *Hanseniaspora uvarum* by agar-well diffusion assay: 1, distilled water; CE extracted by: 2, cool water; 3, hot water; 4, 30% ethanol; 5, 50% ethanol; 6, 80% ethanol; 7, 100% ethanol.

However, as shown in Figure 4a, FGP did not show effective anti yeast activity against *K. servazzii*. To overcome this limitation of FGP, anti yeast activity against *K. servazzii* using natural materials such as ginger, lemon, wasabi, green tea, onion, green onion, radish, citrus extract, and cinnamon was examined. Among them, only cinnamon showed anti yeast activity against *K. servazzii*. Indeed, cinnamon extract has been widely used as a natural food preservative because it contains an abundance of cinnamaldehyde [20,36,37]. Thus, we evaluated the growth inhibitory effect of cinnamon on WCFY, which is known to have excellent antimicrobial activity, especially against yeast. [38]. To optimize the ethanol extraction of the cinnamaldehyde from the cinnamon powder, different concentrations of ethanol [30, 50, 80, and 100% (*v/v*)] were used to produce cinnamon extract in the present study and their anti yeast activity was subsequently evaluated. The results showed that CEE exhibited a wider growth inhibition zone than that of the cinnamon water extract (Figure 5). In particular, CEE extracted with 80% ethanol exhibited strong anti yeast activity against *K. servazzii* (Figure 5a). Furthermore, CEE extracted with 80% ethanol had the highest cinnamaldehyde content (Table 2), which was higher when compared to 100% ethanol. This result indicated that the use of 100% ethanol might reduce the solubility of the cinnamaldehyde, which was similar to the result of a previous report that the extraction efficiency of cinnamaldehyde was increased in around 80% ethanol and then decreased in 100% ethanol [39]. Meanwhile, the cinnamon water extract exhibited lower anti yeast activity against 5 WCFY than the ethanol extracts (Figure 5) and mold appeared over time. Hence, CEE was employed in the subsequent experiments.

### 3.3. Anti Yeast Activity of the FGP and CEE Mixture against WCFY

According to our previous results (Figure 5), given that CEE had an antimicrobial effect against all WCFY strains, it also appeared to be effective in inhibiting white colony formation on kimchi. However, the excessive addition of CEE to kimchi can affect its sensory properties due to its strong flavor. Thus, to effectively suppress white colony formation on the surface of kimchi without adversely affecting the sensory properties of kimchi, both sensory evaluation and anti yeast activity tests were performed using various concentrations of the mixtures of FGP and CEE. The concentration of the garlic powder was determined to be 10% considering the strong smell and clumping that occurred during spraying. First, sensory evaluation was conducted to minimize the sensory influences of a mixture of FGP and CEE as a preservative. The results showed that a mixture of 10% FGP and 1.75% CEE did not significantly affect the appearance, smell, and taste of kimchi compared with that of the control (Table 3). However, a treatment of the 10% FGP and 2% CEE mixture significantly affected the taste of kimchi. Further studies need to be conducted to evaluate the organoleptic characteristic of kimchi with a greater number of panelists because 10 panelists for sensory test is a very limited sample. 

As mentioned above, in fact, it is difficult to apply cinnamon extract directly to foods as a preservative owing to its strong flavor. Thus, studies have recently reported on the method of applying cinnamon extract to a film and using it as a packaging material rather than directly applying cinnamon extract to foods [40,41]. In the future, based on these previous studies, it is necessary to study a method of suppressing white colony formation of kimchi without adverse sensory effects.

**Figure 6:**
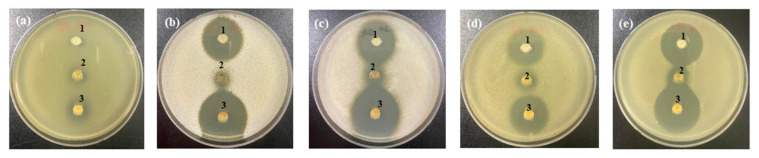
Anti yeast activity of 10% freeze-dried garlic powder (FGP), 1.75% cinnamon ethanol extract (CEE), and the mixture of them against (**a**) *Kazachstania servazzii*, (**b**) *Candida sake*, (**c**) *Debaryomyces hansenii*, (**d**) *Pichia kudriavzevii*, and (**e**) *Hanseniaspora uvarum* by agar-well diffusion assay: 1, 10% FGP; 2, 1.75% CEE; 3, a mixture of 10% FGP and 1.75% CEE.

Thereafter, the anti yeast activity of the mixture was evaluated by the agar-well diffusion assay compared with FGP only (Figure 6). The mixture of 10% FGP and 1.75% CEE exhibited a higher anti yeast activity than that of 10% FGP against all WCFYs including *K. servazzii* (Figure 6a). In conclusion, our results showed that the mixture of 10% FGP and 1.75% CEE is might be suitable for use as a preservative for use inhibiting white colony formation on kimchi. 

### 3.4. Application of the FGP and CEE Mixture on Kimchi

Combining the FGP and CEE mixture with kimchi for the inhibition of white colony formation increases the amount of mixture added, which can affect the sensory properties of kimchi and increase its cost. In addition, generally, white colony was known to form on the surface of kimchi [4]. Thus, in this study, the inhibitory effect of the FGP and CEE mixture on white colony formation on kimchi was determined by spraying the mixture on the surface of the kimchi. Furthermore, 0.02% xanthan gum, a thickening agent, was combined with the FGP and CEE mixture to increase the adhesiveness on the surface of the kimchi, prolonging the inhibitory effect of the mixture. Xanthan gum is permitted as thickening and stabilizing agent in the CODEX standard for kimchi. 

#### 3.4.1. Application of the FGP and CEE Mixture on Kimchi Inoculated WCFY

**Figure 7:**
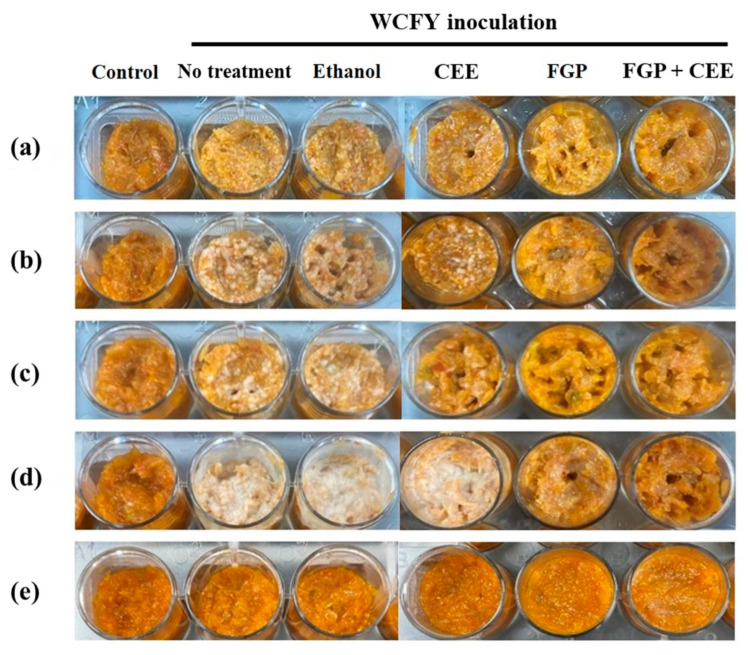
White colony inhibitory effect of 10% freeze-dried garlic powder (FGP), 1.75% cinnamon ethanol extract (CEE), and their mixture on kimchi inoculated with (**a**) *Kazachstania servazzii*, (**b**) *Candida sake*, (**c**) *Debaryomyces hansenii*, (**d**) *Pichia kudriavzevii*, and (**e**) *Hanseniaspora uvarum* during storage at 24 °C for 10 days.

The inhibitory effect of the FGP and CEE mixture on white colony formation on kimchi was evaluated in kimchi inoculated with each WCFY strain (Figure 7). FGP inhibited the growth of *C. sake*, *D. hansenii*, and *P. kudriavzevii* (Figure 7b–d). Moreover, CEE suppressed the growth of *K. servazzii*, *C. sake*, and *D. hansenii* (Figure 7a–c). The FGP and CEE mixture inhibited the growth of all WCFYs. The results indicated that the FGP and CEE mixture demonstrated a growth inhibitory effect on white colony formation during kimchi fermentation and storage. Moreover, as shown in Figure 7, different forms of the white colony were produced on kimchi inoculated with all WCFY strains except for *H. uvarum*. This was probably because *H. uvarum* is not a major WCFY, despite it being isolated from the white colony of kimchi, or because the experimental condition (i.e., 24 °C for 10 d) is not suitable for the growth of this yeast in kimchi. While ethanol is generally known to have excellent antimicrobial activity, it did not exhibit antimicrobial activity against kimchi WCFY (Figure 7a–d). According to previous studies, even though the ethanol tolerance mechanism of yeasts remains elusive, several factors have been reported that may influence ethanol tolerance. For instance, it is reported that an increase in membrane phospholipids and an increase in the content of unsaturated fatty acids, vitamins, and proteins, as well as temperature and osmotic pressure, could contribute to the ethanol tolerance of yeast strains [42,43,44]. Based on the previous studies, it is understood that the innate characteristics and growth environment of WCFY in kimchi might have influenced their ethanol tolerance.

#### 3.4.2. Application of the FGP, CEE, and Xanthan Gum on Kimchi

**Figure 8:**
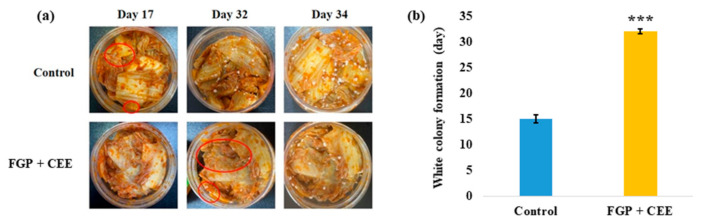
White colony inhibitory effect of the mixture of 10% freeze-dried garlic powder (FGP), 1.75% cinnamon ethanol extract (CEE), and xanthan gum on commercial kimchi during storage at 10 °C for 35 days. (**a**) The images of kimchi formed white colony. The red circle indicated the earliest colony formation. The images shown are representative of three independent experiments. (**b**) The date of the white colony formation on the kimchi surface. Data represent the mean ± SE (*n* = 3); *** (*p* < 0.001) indicates a significant difference vs. the control.

To determine how long the mixture can delay white colony formation on kimchi, kimchi was observed after treatment with a mixture of FGP, CEE, and xanthan gum on the surface during storage at 10 °C. Consequently, white colony formation on kimchi was delayed for an average of 17 d compared to the control kimchi (Figure 8). Most previous studies have focused on microbial community analysis of the white colony in kimchi, its influences on kimchi quality, and safety [4,5,6]. Although there has been a previous study on suppressing WCFY in kimchi using defatted green tea seed extracts [11], this method cannot be used industrially because the manufacturing process is complicated and was not commercialized. Alternatively, our study focused on the method of inhibiting white colony formation in kimchi, and the materials used in this study are expected to be highly practical because they can be easily obtained from the market and the manufacturing method is simple. Therefore, this study can be directly applied in the kimchi industry.

## 4. Conclusions

According to our results (Figure 1, Figure 2 and Figure 3), inhibiting the growth of WCFY by controlling the manufacturing and storage conditions of kimchi seems difficult. Thus, in this study, we evaluated the inhibitory effects of plant-derived natural preservatives against the WCFY of kimchi. FGP exhibited anti yeast activity against *C. sake*, *D. hansenii*, *P. kudriavzevii*, and *H. uvarum* except for *K. servazzii*. Moreover, CEE demonstrated anti yeast activity against *K. servazzii*. The FGP and CEE mixture was employed while considering the anti yeast activity against all WCFYs and the influence on the sensory properties of kimchi. However, since the direct application of the mixture to kimchi may negatively affect the sensory properties of the kimchi, its concentration and amounts are very limited. Therefore, further research on indirect methods such as using the mixture as an anti yeast film coating agent is needed to solve the mentioned problem.

Consequently, the mixture showed a growth inhibitory effect on all WCFYs, and delayed white colony formation on kimchi by an average of 17 d compared to the control kimchi. Therefore, the application of 10% FGP, 1.75% CEE, and 0.02% xanthan gum on kimchi could be an effective way to extend the shelf life of kimchi, especially for export requiring long transportation periods.

## Figures and Tables

**Table 1:** Alliin content measured by liquid chromatography-mass spectrometry (LC/MS).

Sample	Alliin (mg/L)
10% FGP	32.73 ± 4.99 ^c^
20% FGP	63.41 ± 9.16 ^b^
30% FGP	93.38 ± 14.58 ^a^
Raw garlic filtrate	60.43 ± 0.55 ^bc^

^a–c^ Different letters mean significant differences (*p* < 0.05). Values are mean ± standard error (*n* = 3).

**Table 2:** Cinnamaldehyde content measured by high performance liquid chromatography (HPLC).

Cinnamon Extract	Cinnamaldehyde (mg/L)
Extracted by water	581.79
Extracted by hot water	89.22
Extracted by 30% ethanol	2942.29
Extracted by 50% ethanol	9278.27
Extracted by 80% ethanol	11,521.08
Extracted by 100% ethanol	7832.66

**Table 3:** Sensory evaluation of control kimchi and a mixture of freeze-dried garlic powder (FGP) and cinnamon ethanol extract (CEE)-treated kimchi.

Parameter	Control	10% FGP + 1.75% CEE	10% FGP + 2% CEE
Appearance	5.50 ± 1.28 ^a^	4.27 ± 1.55 ^a^	4.64 ± 1.51 ^a^
Smell	4.70 ± 1.79 ^a^	4.40 ± 1.69 ^a^	3.80 ± 0.87 ^a^
Taste	6.00 ± 1.26 ^a^	5.20 ± 1.25 ^ab^	4.40 ± 1.20 ^b^

^a,b^ Different letters within columns denote significant differences (*p* < 0.05). Values are mean ± standard error (*n* = 10).

## Data Availability

The data presented in this study are available in the article.

## References

[1] Lee S.H., Jung J.Y., Jeon C.O. (2015). Source tracking and succession of kimchi lactic acid bacteria during fermentation. J. Food Sci..

[2] Jung J.Y., Lee S.H., Jeon C.O. (2014). Kimchi microflora: History, current status, and perspectives for industrial kimchi production. Appl. Microbial. Biotechnol..

[3] Moon S.H., Chang M., Kim H.Y., Chang H.C. (2014). *Pichia kudriavzevii* is the major yeast involved in film-formation, off-odor production, and texture-softening in over-ripened Kimchi. Food Sci. Biotechnol..

[4] Suzuki A., Muraoka N., Nakamura M., Yanagisawa Y., Amachi S. (2018). Identification of undesirable white-colony-forming yeasts appeared on the surface of Japanese kimchi. Biosci. Biotechnol. Biochem..

[5] Kim J.Y., Kim J., Cha I.T., Jung M.Y., Song H.S., Kim Y.B., Lee C., Kang S.Y., Bae J.W., Choi Y.E. (2019). Community structures and genomic features of undesirable white colony-forming yeasts on fermented vegetables. J. Microbiol..

[6] Kim M.J., Lee H.W., Kim J.Y., Kang S.E., Roh S.W., Hong S.W., Yoo S.R., Kim T.W. (2020). Impact of fermentation conditions on the diversity of white colony-forming yeast and analysis of metabolite changes by white colony-forming yeast in kimchi. Food Res. Int..

[7] Martorell P., Stratford M., Steels H., Fernández-Espinar M.T., Querol A. (2007). Physiological characterization of spoilage strains of *Zygosaccharomyces bailii* and *Zygosaccharomyces rouxii* isolated from high sugar environments. Int. J. Food Microbial..

[8] Pérez-Díaz I.M., McFeeters R.F. (2010). Preservation of acidified cucumbers with a natural preservative combination of fumaric acid and allyl isothiocyanate that target lactic acid bacteria and yeasts. J. Food Sci..

[9] Shwaiki L.N., Arendt E.K., Lynch K.M., Thery T.L. (2019). Inhibitory effect of four novel synthetic peptides on food spoilage yeasts. Int. J. Food Microbiol..

[10] Pérez-Díaz I.M. (2011). Preservation of acidified cucumbers with a combination of fumaric acid and cinnamaldehyde that target lactic acid bacteria and yeasts. J. Food Sci..

[11] Yang E.J., Seo Y.S. (2017). Stability of anti-yeast activities and inhibitory effects of defatted green tea seed extracts on yeast film formation. J. Korean Soc. Food Sci. Nutr..

[12] Xu W., Qu W., Huang K., Guo F., Yang J., Zhao H., Luo Y. (2007). Antibacterial effect of grapefruit seed extract on food-borne pathogens and its application in the preservation of minimally processed vegetables. Postharvest Biol. Technol..

[13] Kim Y.S., Kyung K.H., Kim Y.S. (2000). Inhibition of soy sauce film yeasts by allyl isothiocyanate and horse-radish powder. Korean J. Food Nutr..

[14] Kim H.J., Semeneh S., Ammara A., Kim B.J., Park J.G., Kang S.N. (2020). Antioxidant and antimicrobial activity in oat (*Avena sativa* L.) leaf extracts and its effect on the characteristics of emulsion sausage. J. Agri. Life Sci..

[15] Hu F., Tu X.F., Thakur K., Hu F., Li X.L., Zhang Y.S., Zhang J.G., Wei Z.J. (2019). Comparison of antifungal activity of essential oils from different plants against three fungi. Food Chem. Toxicol..

[16] Choi Y.J., Jin H.Y., Yang H.S., Lee S.C., Huh C.K. (2016). Quality and storage characteristics of yogurt containing *Lacobacillus sakei* ALI033 and cinnamon ethanol extract. J. Anim. Sci. Technol..

[17] Sallam K.I., Ishioroshi M., Samejima K. (2004). Antioxidant and antimicrobial effects of garlic in chicken sausage. LWT-Food Sci. Technol..

[18] Nazari M., Ghanbarzadeh B., Kafil H.S., Zeinali M., Hamishehkar H. (2019). Garlic essential oil nanophytosomes as a natural food preservative: Its application in yogurt as food model. Colloid Interface Sci. Commun..

[19] Leontiev R., Hohaus N., Jacob C., Gruhlke M.C., Slusarenko A.J. (2018). A comparison of the antibacterial and antifungal activities of thiosulfinate analogues of allicin. Sci. Rep..

[20] Balaguer M.P., Lopez-Carballo G., Catala R., Gavara R., Hernandez-Munoz P. (2013). Antifungal properties of gliadin films incorporating cinnamaldehyde and application in active food packaging of bread and cheese spread foodstuffs. Int. J. Food Microbiol..

[21] Kurtzman C.P., Robnett C.J. (1988). Identification and phylogeny of ascomycetous yeasts from analysis of nuclear large subunit (26S) ribosomal DNA partial sequences. Antonie van Leeuwenhoek.

[22] Zhu Q., Kakino K., Nogami C., Ohnuki K., Shimizu K. (2016). An LC-MS/MS-SRM method for simultaneous quantification of four representative organosulfur compounds in garlic products. Food Anal. Methods.

[23] Helal A., Tagliazucchi D., Verzelloni E., Conte A. (2014). Bioaccessibility of polyphenols and cinnamaldehyde in cinnamon beverages subjected to in vitro gastro-pancreatic digestion. J. Funct. Foods.

[24] Cizeikiene D., Juodeikiene G., Paskevicius A., Bartkiene E. (2013). Antimicrobial activity of lactic acid bacteria against pathogenic and spoilage microorganism isolated from food and their control in wheat bread. Food Control.

[25] Zhang T., Jeong C.H., Cheng W.N., Bae H., Seo H.G., Petriello M.C., Han S.G. (2019). Moringa extract enhances the fermentative, textural, and bioactive properties of yogurt. LWT-Food Sci. Technol..

[26] Choi Y.M., Whang J.H., Kim J.M., Suh H.J. (2006). The effect of oyster shell powder on the extension of the shelf-life of kimchi. Food Control.

[27] Park S.J., Park K.Y., Jun H.K. (2001). Effects of commercial salts on the growth of kimchi-related microorganisms. J. Korean Soc. Food Sci. Nutr..

[28] Meng X., Lee K., Kang T.Y., Ko S. (2015). An irreversible ripeness indicator to monitor the CO_2_ concentration in the headspace of packaged kimchi during storage. Food Sci. Biotechnol..

[29] Lee J.S., Jeong S., Lee H.G., Cho C.H., Yoo S. (2018). Development of a sulfite-based oxygen scavenger and its application in Kimchi packaging to prevent oxygen-mediated deterioration of Kimchi quality. J. Food Sci..

[30] Jang H.J., Lee H.J., Yoon D.K., Ji D.S., Kim J.H., Lee C.H. (2018). Antioxidant and antimicrobial activities of fresh garlic and aged garlic by-products extracted with different solvents. Food Sci. Biotechnol..

[31] Oh T.Y., Kyung K.H. (2011). Isolation and purification of garlic specific organic compounds. Korean J. Food Sci. Technol..

[32] Lawson L.D., Wang Z.J. (2001). Low allicin release from garlic supplements: A major problem due to the sensitivities of alliinase activity. J. Agri. Food Chem..

[33] Marchese A., Barbieri R., Sanches-Silva A., Daglia M., Nabavi S.F., Jafari N.J., Lzadi M., Ajami M., Nabavi S.M. (2016). Antifungal and antibacterial activities of allicin: A review. Trends Food Sci. Technol..

[34] Kim J.Y., Lee Y.C., Kim K.S. (2002). Effect of heat treatments on the antimicrobial activities of garlic (Allium sativum). J. Microbiol. Biotechnol..

[35] Chae S.K. (2007). Studies on the changes in the alliinase activity during the drying of garlic. Korean J. Sanitation..

[36] Abdel-Mallek A.Y., Bagy M.M.K., Hasan H.A.H. (1994). The in vitro anti-yeast activity of some essential oils. J. Islam. Acad. Sci..

[37] Shreaz S., Sheikh R.A., Bhatia R., Neelofar K., Imran S., Hashmi A.A., Manzoor N., Basir S.F., Khan L.A. (2011). Antifungal activity of α-methyl trans cinnamaldehyde, its ligand and metal complexes: Promising growth and ergosterol inhibitors. Biometals.

[38] Makwana S., Choudhary R., Dogra N., Kohli P., Haddock J. (2014). Nanoencapsulation and immobilization of cinnamaldehyde for developing antimicrobial food packaging material. LWT-Food Sci. Technol..

[39] Kim N.M., Yang J.W., Kim W.J. (1993). Effect of ethanol concentration on index components and physicochemical characteristics of cinnamon extracts. Korean J. Food Sci. Technol..

[40] Wen P., Zhu D.H., Wu H., Zong M.H., Jing Y.R., Han S.Y. (2016). Encapsulation of cinnamon essential oil in electrospun nanofibrous film for active food packaging. Food Control..

[41] Khumkomgool A., Saneluksana T., Harnkarnsujarit N. (2020). Active meat packaging from thermoplastic cassava starch containing sappan and cinnamon herbal extracts via LLDPE blown-film extrusion. Food Packag. Shelf Life.

[42] D’amore T., Stewart G.G. (1987). Ethanol tolerance of yeast. Enzyme Microb. Technol..

[43] Alexandre H., Rousseaux I., Charpentier C. (1994). Ethanol adaptation mechanisms in *Saccharomyces cerevisiae*. Biotechnol. Appl. Biochem..

[44] You K.M., Rosenfield C.L., Knipple D.C. (2003). Ethanol tolerance in the yeast *Saccharomyces cerevisiae* is dependent on cellular oleic acid content. Appl. Environ. Microbiol..

